# Patients’ acceptability of a patient-reported outcome measure in cardiac rehabilitation (the PRO-Heart-DK)—a mixed methods study using the Theoretical Framework of Acceptability

**DOI:** 10.1186/s41687-024-00831-8

**Published:** 2025-03-25

**Authors:** Emma Dedic, Heidi Sønderby Vistisen, Ann-Dorthe Zwisler, Bente Faurby Pedersen, Karin Lundsby Kappel, Helle Kanstrup, Rikke Elmose Mols, Cecilie Lindström Egholm

**Affiliations:** 1 REHPA, The Danish Knowledge Centre for Rehabilitation and Palliative Care, Odense University Hospital, Nyborg, Denmark; 2 Department of Clinical Research, University of Southern Denmark, Odense, Denmark; 3 Department of Cardiology, Aarhus University Hospital, Aarhus N, Denmark; 4 Department of Cardiology, Odense University Hospital, Odense, Denmark; 5 Rehabilitation and Palliative Care Research Group, Department of Oncology, Rigshospitalet-Copenhagen University Hospital, Copenhagen, Denmark; 6 Department of Clinical Medicine, Faculty of Health and Medical Sciences, University of Copenhagen, Copenhagen, Denmark; 7 Nordfyn Municipality, Søndersø, Denmark

**Keywords:** Patient-reported outcome measure, Cardiac rehabilitation, Ischemic heart disease, Acceptability, Theoretical Framework of Acceptability, Patient engagement, Patient management

## Abstract

**Background:**

The integration of Patient Reported Outcome Measures (PROM) in cardiac rehabilitation practice has potential to enhance patient involvement and management. User acceptance is crucial for successful implementation of healthcare interventions, but limited literature addresses PROM acceptability among cardiovascular patients. This study explored the acceptability of a new national PROM in cardiac rehabilitation clinical practice for patients with ischemic heart disease (IHD) in Denmark.

**Methods:**

Patients who responded to the PROM were invited to complete two brief surveys evaluating perceived relevance, usefulness, and satisfaction. A purposefully selected subsample participated in semi structured interviews to gather in-depth experiences. A parallel convergent mixed-methods design was used with the Theoretical Framework of Acceptability applied to structure and interpret findings.

**Results:**

105 and 119 patients respectively responded to the two evaluation surveys (response-rates 56.5% and 53.4% respectively) and 25 patients were interviewed. The study showed a strong willingness to engage with PROMs, indicating a high overall acceptability. Most patients perceived the PROM helpful for preparation and enhancing communication during consultations. A minority of patients reported emotional reactions and experienced issues with questionnaire comprehensiveness, structure, and relevance.

**Conclusion:**

The findings indicate that most IHD patients find PROM relevant and useful in cardiac rehabilitation. To enhance acceptability and, hence, future implementation, improvements are needed in clinical settings by providing adequate patient information, effectively using PRO results, and addressing patients’ emotional reactions. Additionally, PROM development should focus on ensuring the questionnaire’s relevance, comprehensiveness, and structure.

**Supplementary Information:**

The online version contains supplementary material available at 10.1186/s41687-024-00831-8.

## Abstract (plain language summary)

Patient-reported outcome measures (PROM) are increasingly applied in clinical settings in order to enhance patient involvement and patient management. The use of PROM in practice is, like any other healthcare intervention, depending on the patients’ accept. Applying survey and interview as methods, this study explored patients’ acceptability of a PROM in cardiac rehabilitation clinical practice using the Theoretical Framework of Acceptability. Overall, the findings indicate that many patients with ischemic heart disease consider PROM to be relevant and useful as part of the cardiac rehabilitation process which suggest high level of acceptability. However, some aspects of the PROM, specifically the perceived questionnaire relevance, comprehensiveness and structure (e.g. lack of suitable response-options) need further improvement to enhance patients’ acceptability and ensure their future engagement.

**Supplementary Information:**

The online version contains supplementary material available at 10.1186/s41687-024-00831-8.

## Background

Cardiac rehabilitation is a comprehensive intervention [1, 2] which aims to improve the functional level and health-related quality of life of cardiovascular patients, and reduce the risk of disease progression [3]. Recently, several European cardiovascular organizations jointly recommended incorporating patient-reported outcomes in cardiac rehabilitation clinical practice to enhance patient involvement and management [4]. Patient-reported outcomes (PRO) is defined as any health status reports coming directly from the patient [5] and this information (PRO-data) is collected via standardized generic and/or disease specific questionnaires (PROM). PROMs aim to evaluate patients’ self-reported health perceptions and can help healthcare professionals (HCPs) to identify underreported and unrecognized problems [6, 7]. Furthermore, PROMs have shown potential to improve communication between patients and HCPs [8–10], enhance diagnosis and disease management, and achieve incremental improvements in patients’ quality of life [9]. Finally, PROMs are increasingly used as outcome measure in clinical practice [11–13].

Internationally, various initiatives have focused on developing and implementing PROMs for cardiovascular disease [14] and for cardiac rehabilitation in particular, e.g. in Great Britain [15, 16], Switzerland [17] and through the U.S. based International Consortium for Health Outcomes Measurement initiative [18], all emphasizing the application of PROMs in clinical practice for monitoring and improvement of cardiovascular prevention. In Denmark too, significant efforts have been made to develop and implement PROMs [19], including a PROM for cardiac rehabilitation in patients with ischemic heart disease (IHD) (hereafter ‘PRO-Heart-DK’), which was developed under the auspices of the Danish Health Data Authority through a participatory process with patients and HCPs [20]. The primary aim of developing the PRO-Heart-DK was to harness potential benefits of PROMs and ensure their standardized use across cardiac rehabilitation clinical settings to enhance quality of care. The standardized, national PRO-Heart-DK was launched for implementation after a feasibility test period.

Research suggests that the implementation, uptake, adherence, and effectiveness of health interventions are significantly influenced by their acceptability among patients and healthcare professionals [21–23]. As conceptualized in the Theoretical Framework of Acceptability, acceptability reflects the perceptions of individuals delivering or receiving an intervention based on their cognitive and emotional responses to the intervention [24]. Interventions deemed unacceptable by those delivering or receiving them face challenges in implementation and scale-up due to lack of engagement [23]. Therefore, acceptability is recognized as a critical facet in designing and evaluation of complex health interventions [25].

Although ongoing international initiatives aim to integrate PROMs into cardiac rehabilitation to enhance patient involvement and clinical outcomes [4], limited research has explored the acceptability of PROMs in this setting. Two studies demonstrated good acceptability when PROMs were used at the start and end of cardiac rehabilitation programmes [15, 26], reporting that patients were able to complete the questionnaires independently at home [15] and found them relatively easy to fill in [26]. However, evaluations of the acceptability of PROMs in Danish cardiac rehabilitation settings from the patients’ perspective are still lacking, leaving gaps in the understanding of their uptake and overall effectiveness. Assessing patients’ acceptability of PROMs is thus crucial because it directly influences their willingness to engage with and adhere to these measures, ultimately impacting the intervention’s success [15, 27]. Aligned with the international call for increased application of PROM into cardiac rehabilitation practices, this study aimed to explore acceptability of the new PRO-Heart-DK form the patients’ perspective, including their perceptions of its comprehensibility, usability, relevance, and thus value. By focusing on assessing patient perspectives, this research seeks to support the implementation of PROM in cardiac rehabilitation practice and ultimately to enhance the quality of cardiac rehabilitation to the benefit of the patients.

## Materials and methods

This study was nested in the Implementation of Patient Reported Outcomes via questionnaires in Cardiac Rehabilitation (IMPROveCARE) study; a larger feasibility project evaluating implementation of the PRO-Heart-DK. We applied a mixed methods approach to assess the patients’ acceptability of this new PROM, combining two brief evaluation surveys and interview data. A convergent parallel design was chosen [28] and we followed COREQ guidelines [29].

### Setting

In Denmark, specialized cardiac rehabilitation services are organized at hospital level while general cardiac rehabilitation is the responsibility of the municipalities. The project was conducted across two large university hospitals and four (out of 98) municipalities in three of Denmark’s five healthcare regions. These sites volunteered to take part in the feasibility test and as part of this, they were required to integrate the PRO-Heart-DK into their local care processes using existing IT-systems. Adult patients (+ 18 years) with IHD were asked to complete the PRO-Heart-DK electronically at home prior to both initial hospital consultation and final municipal consultation. The questionnaires were distributed via secure email or electronic patient systems. Patients who were not signed up to secure mail/electronic patient systems were not able to participate except for at three sites who provided a local opportunity to respond on an iPad in the waiting room or with help from staff. Prior to responding, patients were informed in writing that their response would be utilized by HCPs during consultations.

The PRO-Heart-DK comprises 76 items focusing on symptoms, mental well-being, functional status, health-related quality of life, coping, social support, and risk factors regarding smoking, exercise, diet and alcohol. The items are a combination of generic and disease specific items, validated scales/questionnaires, and in-house made items [19]. An overview of the content is provided in Appendix 1. The primary purpose of PRO-Heart-DK is to be used in clinical visits to support patient-HCP communication and disease management, and when filled out more than once during the cardiac rehabilitation programme, to measure outcomes.

### Data collection

#### Evaluation surveys

All patients who responded to the PRO-Heart-DK were invited to participate in two evaluation surveys.

**Fig. 1:**
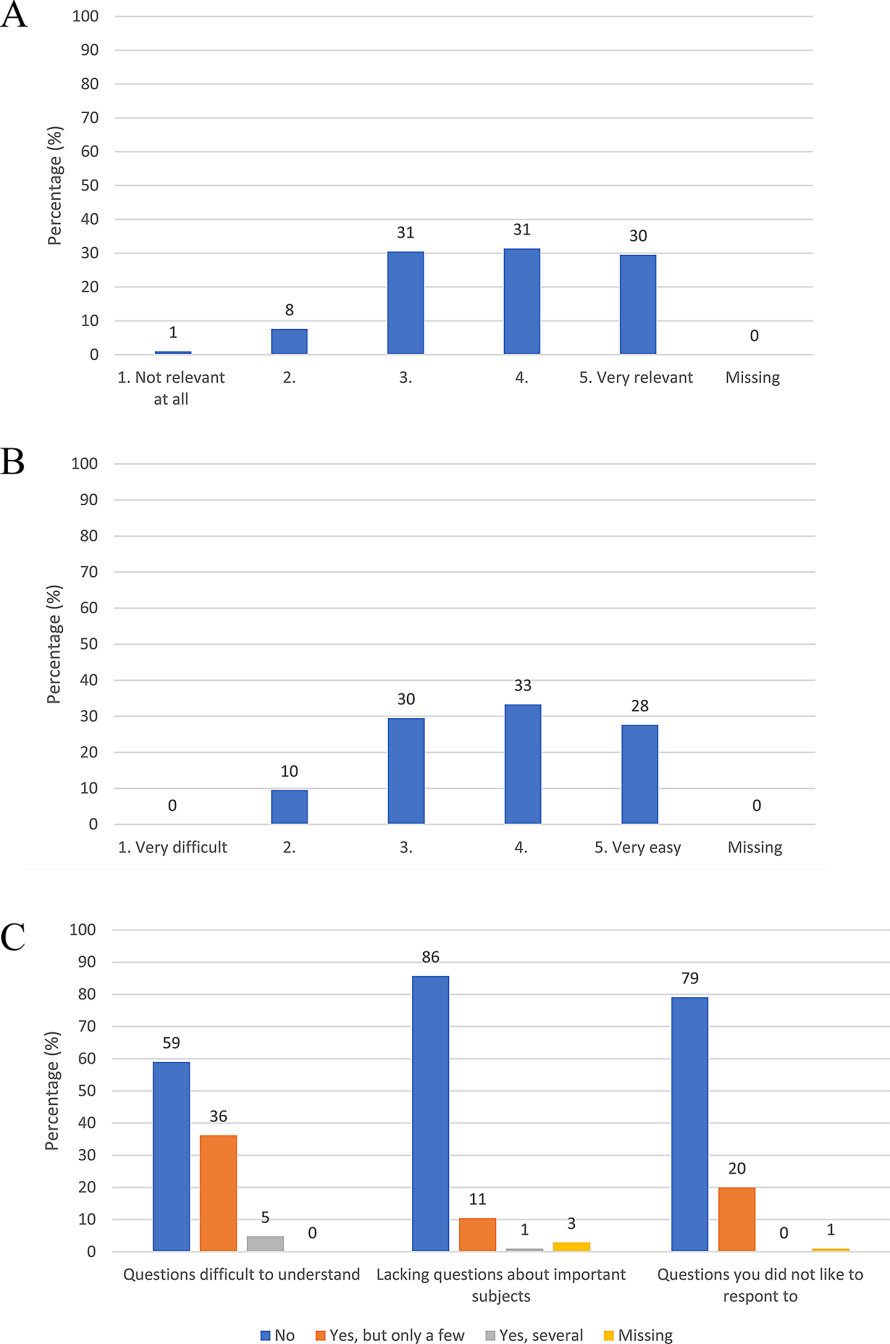
**A** Responses to the PRO Evaluation Questionnaire (*n* = 105), item 1 ’’How relevant were the questions for your cardiac rehabilitation process?’’ **B** Responses to the PRO Evaluation Questionnaire (*n* = 105), item 2 “How easy/difficult was it for you to respond to the questionnaire?’’ **C** Responses to the PRO Evaluation Questionnaire (*n* = 105), item 3–5

**Fig. 2:**
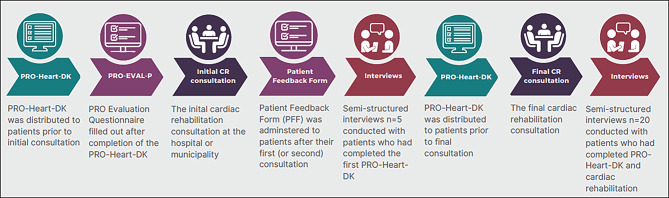
Illustration of the data collection

**Fig. 3:**
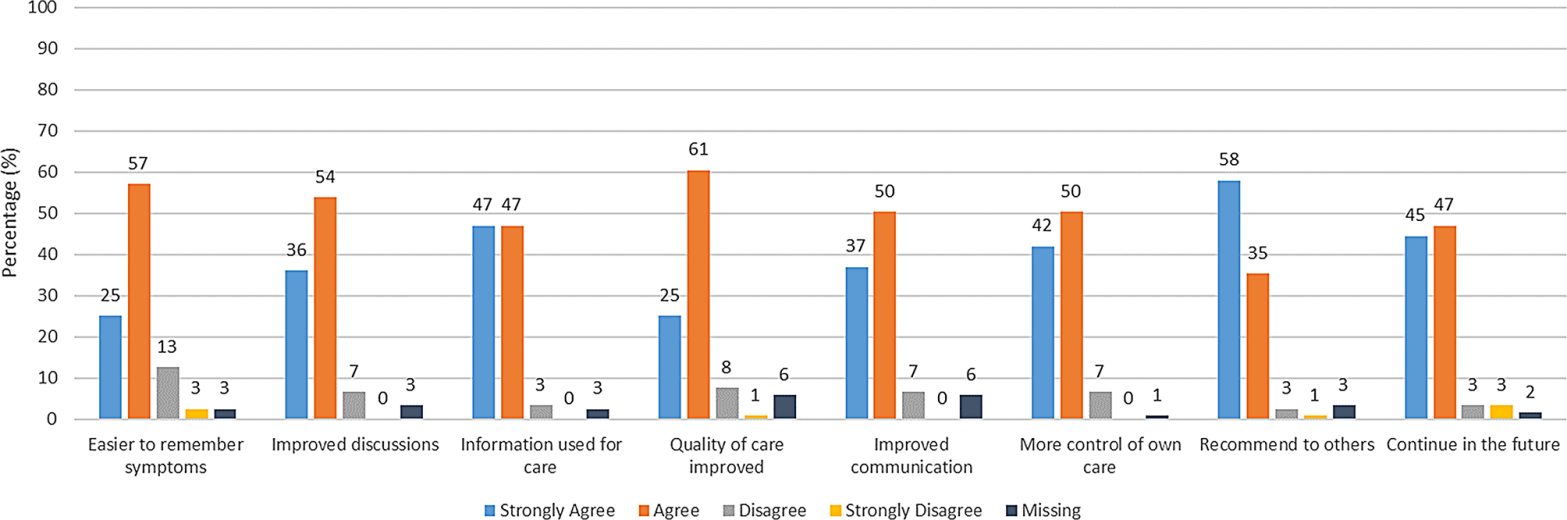
Responses to the Patient Feedback Form (PFF) (*n* = 119)

*The PRO Evaluation Questionnaire* (PRO-EVAL-P) measures the perceived relevance of a PROM from the perspective of patients. Initially developed for evaluation of a PROM for diabetes [30] it was adapted for the purpose of the IMPROveCARE study: wording related to diabetes was changed to wording related to cardiac rehabilitation. It consisted of five items with focus on the perceived relevance and ease of responding to the PROM (Fig. 1a, b and c and Appendix 2) with response options on 3- or 5-point Likert scales. The PRO-EVAL-P was distributed electronically to collect feedback immediately after filling out the initial PRO-Heart-DK (Fig. 2). *The Patient Feedback Form* (PFF) measures patient satisfaction and can be used to evaluate the usefulness and value of a PROM from the patient perspective [31]. A translated, culturally adapted, and validated version of the PFF was administered electronically or on paper (depending on local IT-systems and care processes) to the patients after the PRO-Heart-DK had been filled out and discussed in a cardiac rehabilitation consultation (Fig. 2). This version consisted of eight generic items regarding patients’ experiences, e.g. whether PRO prepared them, was used in the consultation, improved their involvement, quality of care, and communication (Fig. 3 and Appendix 2). The patients evaluated their level of agreement on a scale with four response options (‘strongly agree’ to ‘strongly disagree’).

#### Semi-structured interviews

A subset of patients were invited to participate in an individual interview. Invitations were based on a principle of maximum variation. All interviews were conducted within one week after a consultation with the HCP (Fig. 2). The interviews were conducted between December 2019 and October 2020 by two of the authors in the patients’ home or by telephone in accordance with patient preferences at the time of COVID-19 social distancing. A semi-structured interview guide was designed based on existing knowledge covering various aspects of PROMs, including perceived purpose, complexity, application, patient involvement, relevance, and meaningfulness. All interviews were audio recorded and transcribed verbatim.

### Data analyses

We analyzed the two types of data separately and integrated results during interpretation [28]. Descriptive statistics were applied to summarize survey data into numbers and percentages. The interview data were analyzed in a two-step process. First, data were thematically analyzed using inductive thematic analysis approach [32], using NVivo software. This approach allows themes to be developed from data without preconceived concepts [32]. Four out of six iterative phases were followed in thematic analysis process [32] until subthemes were developed. The analytical process is described in Appendix 3. Secondly, the identified subthemes were deductively organized according to the constructs in the Theoretical Framework of Acceptability.

#### Theoretical framework of acceptability (TFA)

The TFA emphasizes that acceptability is a multifaceted construct that reflects the extent to which people delivering or receiving a healthcare intervention consider it to be appropriate based on anticipated or experienced cognitive and emotional responses to the intervention [24]. TFA comprises seven constructs: affective attitude, burden, ethicality, intervention coherence, opportunity cost, perceived effectiveness, and self-efficacy. The framework can be applied for evaluation of healthcare interventions prospectively, concurrently and retrospectively [24].

In the present study, the patients’ acceptability of the PRO-Heart-DK was assessed retrospectively.

#### Integration of data using TFA

We organized both the quantitative survey data and the qualitative subthemes according to the TFA constructs to get a theory based and structured understanding of patients’ acceptability of the PRO-Heart-DK [28]. A description of how we interpreted the constructs and organized data is presented in Appendix 4. Analytic rigor in the analysis process was ensured through analyst triangulation [33]. Five interviews were double coded by first and the last author, remaining authors acted as critical peers.

## Results

The PRO-EVAL-P survey was distributed to 186 patients and we received responses from 105 (response-rate 56.5%). Seventy-seven (73%) were men, with a mean age of 62.2 years (standard deviation (SD) 11.9 years). The PFF survey was filled out by 119 of 223 respondents (response-rate 53.4%), including 84 (71%) men, with a mean age of 66.6 years (SD 11.2). Semi-structured interviews were conducted with 25 participants who were patients with IHD and had completed one or more PROM questionnaires during their cardiac rehabilitation process. They lasted between 20 and 60 min. Characteristics of interviewees are presented in Table 1.

**Table 1:** Interview participants’ characteristics

Cluster*	Cluster a	Cluster b	Cluster c	Total
**Gender**				
*Female*	2	3	4	9
*Male*	2	6	8	16
**Age**				
(years, range)	75–80	57–80	34–82	34–82
**Civil status**				
*Living alone*	0	4	2	6
*Living with someone*	4	5	10	19
**Education**				
*Elementary school*	2	1	4	7
*Upper secondary/ vocational school*	1	5	7	13
*Higher education*	1	3	1	5

*Cluster: Clusters refer to the hospitals and municipalities within a healthcare region

### Overall acceptability

A vast majority, 93% of respondents to the PFF expressed their readiness to recommend completing the PRO-Heart-DK questionnaire to other patients and 92% agreed to continue responding in the future (Fig. 3), suggesting a high level of overall acceptability.

In line with the survey results, the interviewees collectively perceived the use of PROM in cardiac rehabilitation as valuable and expressed a willingness to participate again. Their motivation stemmed from a belief that PROM could enhance IHD treatment and improve healthcare overall. The interviewees’ reports reflected an altruistic motivation to contribute.

Yeah, I think it’s okay. I mean, I’d like to contribute. When I have received help myself, I would like to contribute. I would be willing to do that so it can benefit other people. Male, 75 y.

### Affective attitude

In the PRO-EVAL-P survey, 79% of participants were comfortable with the questions in the PRO-Heart-DK, whereas 20% expressed discomfort or dislike towards certain questions (Fig. 1c). This dissatisfaction indicated that a subset of participants experienced negative reactions, while completing the questionnaire.

The interviews revealed a spectrum of different emotional reactions. The positive feedback included reports that the use of PROM provided a sense of safety and made individuals feel at the center of their care. These patients commented that questionnaires were a good initiative that contributed to well-being.

Yes, but I definitely think it was optional whether one wants to fill it out or not. I only think positively about it. It was yet another good initiative, yet another good offer. Male, 67 y.

Conversely, some participants found the process distressing, particularly when lifestyle responses were discussed with healthcare professionals. For these interviewees, the questionnaire brought attention to their lifelong health issues, evoking a feeling of being held accountable for their current health circumstances. The situation was also perceived as vulnerable and discouraging, especially when faced with alarming or concerning results.

You turn yourself inside out a little, and you could of course omit answering it correctly, but then it wouldn’t be useful at all, if you are not honest in relation to the questionnaire. And always—for someone like me—weight, exercise and things like that, that you should, it’s always like getting a bucket of water in your head when you have to answer it.” Female 70, y

Others expressed frustration about being unfairly judged, feeling that their overall efforts toward a healthy lifestyle were overshadowed by minor dietary habits.

Then she [the HCP] said that I scored a little high in the red zone on diet. I lay my cards on the table—I do eat dessert and whipped cream, and when you think about all the healthy things you eat, it doesn’t really show. But eating a little wrongly now and then pops up right away. Female, 62 y.

For some, hereditary factors added to their sense of frustration.

I agree that I have a big responsibility to manage my illness and adjust my lifestyle. At the same time, I don’t have a lifestyle that should cause heart disease. I have hereditary traits that led to it, so I can’t do much to change my situation. Female, 64 y.

Some participants experienced sadness, particularly related to loss of sexual function due to age and/or illness.

I thought it was very personal, because it has been there and now, I don’t have it anymore, so there is sadness about that, right? Female, 80 y.

Further, the questions about sexual problems/intimacy were intrusive and upsetting for some.

### Burden

Among respondents in the PRO-EVAL-P survey, 61% reported it being easy or very easy to respond to the PRO-Heart-DK questionnaire, while 10% expressed difficulty, signifying that one out of ten of participants encountered difficulties engaging in PROM (Fig. 1b).

The interviews revealed a range of different perspectives regarding the burden of responding to the PRO-Heart-DK., some participants found the questionnaire straightforward and accessible.

So, for me, thought the questions were easy and easily understandable. They were presented in a nice way (…) and I also think the questionnaire was very straightforward Female, 34 y.

It went fine. Despite my old age, I had no trouble sitting down and filling it out online. Male, 77 y.

However, others highlighted challenges, particularly related to its length and repetitive structure.

It wasn’t just a single page; it was many pages. A lot of pages with many questions. It was quite extensive. Male, 80 y.

Some also struggled with unclear or poorly worded items.

I thought there were some really bad questions overall (…) Especially about diet. Female, 62 y.

### Ethicality

No survey data were related to this construct. In the interviews, it became evident that the inclusion of the topic addressing challenges related to intimacy/sex within the PROM elicited a variety of reactions. Some interviewees expressed surprise at its inclusion, some found it informative, and others considered it too personal or irrelevant, indicating that this topic did not fit with all interviewees’ personal values.

There were some [questions] I didn’t think were that relevant. It was that intercourse question. I didn’t think that was their concern. But of course, I answered. Male, 57 y.

I want to say, the thing with sexuality, the thing with sex, well it was actually surprising to me that it could also be a side effect of having a heart disease. Female, 34 y.

There was also a perception that the questionnaire’s pre-determined response-options were too rigid, which hindered the provision of a nuanced description of their complex situations and experiences, e.g. related to diet, physical symptoms and pain.

The thing about being asked if you have chest pain and you say yes, I have. You have some pain, but you do not know if it is muscular or the other. (…) I’ve been living with it for four years and I’ve felt a bit tightening in my chest. But what kind of pain do I have to explain? I find it difficult to explain in the questionnaire. Are you in pain? Pain on the scale from 1 to 10 for example, right. Female, 61 y.

Because of the perceived lack of suitable response-options, some patients mentioned that they replied randomly and thus perceived the questionnaire as not being true and fair, which contributed to a reluctance to respond. These interviewees suggested adding comment boxes to the questions so they can add information not captured by the standard response-options. A few stated that they would have preferred not using questionnaires at all.

Maybe it would have been better if someone called and talked to me about it instead. Female, 61 y.

### Intervention coherence

No survey data were related to this construct. The interviewees had varied assumptions and understanding about the purpose of the PRO-Heart-DK. Despite written information provided with the invitation to complete the questionnaire, there existed a discrepancy in how well-informed interviewees felt about its purpose.

I almost assumed that it was part of my treatment. Male, 72 y.

A few of the interviewees who had received verbal reminders to complete the questionnaire were more aware of how data were to be used in their rehabilitation process.

Because I had been informed that when I arrived for that control visit, the first one, there would indeed be a discussion about diet and how one is feeling, as one feels. I knew. I was informed. Male, 74 y.

Others assumed that the PROM would be used to improve cardiovascular disease treatment and healthcare in general. Some filled out the PRO-Heart-DK because they were asked to and/or did it for the healthcare professionals’ sake, not attributing any personal value to it.

I actually did not ponder that much. I have reasonable confidence in the healthcare system, that they will use it where it is needed. Male, 78 y.

There may be some value in it, but I do not really know what to use it for […] So, I answer those questions to help you, if you can use it for anything. Male, 57 y.

### Opportunity cost

Interviewees reported that they spent 15–30 minutes to complete the questionnaire, for most it took 15–20 minutes. Some found this reasonable, others found it too long. Some interviewees emphasized that the only investment was the time contributed, including time spent by those who assisted in responding, e.g. a spouse or child.

It doesn’t take oceans of time; the questionnaire is a good idea Female, 70 y.

It was quite tricky to get the form in the e-box; I had to try for several days before succeeding Female, 56 y.

It took an enormously long time. Female, 80 y.

Most interviewees had access to electronic devices at home, but one patient sought help from his neighbor.

Several interviewees expressed that completing the questionnaire was a good idea as long as the information they provided was utilized effectively. However, if their data were unused, it would be seen as a waste of time for everyone involved.

### Perceived effectiveness

The majority of respondents acknowledged in the PFF survey several benefits from filling out the PROM. They reported improvements in remembering symptoms (82%), enhanced communication (87%), better discussions with healthcare professionals (90%), improved quality of care (86%), and a heightened sense of control over their own care (92%) (Fig. 3). Notably, 96% indicated that their data were used actively during cardiac rehabilitation consultations.

In the PRO-EVAL-P survey the majority of respondents (91%) found the questions in the PRO-Heart-DK relevant for their rehabilitation process. However, a minority (9%) disagreed (Fig. 1a). Notably, 12% of participants commented that certain questions addressing crucial aspects of their life with cardiovascular disease were missing (Fig. 1c), suggesting that the questionnaire did not resonate with all patients.

For some interviewed patients, the PRO-Heart-DK served as a preparation tool, setting the agenda for consultations by prompting discussions on various topics and requiring them to take a stance. Filling out the PRO-Heart-DK was also viewed as an opportunity for self-reflection, revision of lifestyle, and self-expression. It established a common starting point, fostered more sincere and open conversations, and created a sense of being acknowledged, heard, and actively involved in their care.

Well, I actually think it’s really nice to be asked and get involved in your own treatment. I think that is really positive in principle. I think it’s a good idea […] that one gets to increase one’s awareness about things, by being asked to answer those questions. Female, 64 y.

In the interviews, topics encompassing lifestyle, diet, mental health, and overall well-being were considered most relatable and hence valuable. Filling out the questionnaire provided valuable insights into the IHD, aiding in identifying potential psychological issues and addressing concerns about the likelihood of a new cardiac incident, empowering patients to address these aspects during cardiac rehabilitation consultations.

And then there were also some questions about how I experienced it mentally and I thought it was very relevant for me. (…) I actually had a cardiac arrest, and it has been impressed in my head for a while. It has probably also given me a certain, maybe a little bit of anxiety, so I am somewhat anxious at some points. And I think that I had the opportunity to come forth with that in this context. Male, 78 y.

And it is relevant in relation to what has happened, so I think it is very relevant. So, the whole thing, also in relation to exercise, because you know that activity is good for the heart and good for everything. Female, 70 y.

Among some interviewed patients with long-term heart conditions, the questionnaire topics appeared less relevant. These individuals believed they possessed the necessary knowledge and skillset to manage their condition effectively. Thus, they perceived the questionnaire as redundant or less informative for their specific situation.

No. Not new things. I knew it has something to do with cholesterol, and I knew it. And the blood pressure, I was told, at least for the first time, ten years ago. So, it hasn’t given me anything new, I wouldn’t say that. Male, 80 y.

There were patients who expressed that the questionnaire neither prepared them nor added new knowledge. Some said they preferred an opportunity to ask individual questions, which made the conversation experience with HCPs positive in their opinion.

In contrast to the survey results, interviewees expressed varied perceptions regarding how their answers were approached and used by HCPs. Some patients reported their data were undoubtedly discussed, which made them feel involved in their care.

The first [PRO-Heart-DK], the nurse at the hospital used it during the interview I was at, and the physiotherapist also used it here at the final interview […] she also did a final test on me, where she compared how I had performed compared to when I started, so she used the questionnaire actively. Female, 61 y.

When PROM responses were not mentioned during consultations, it was interpreted as a lack of follow-up, leading to a sense of unmet expectations.

I probably thought that he [the doctor] would review the questionnaire and then based on that have a conversation about how I felt. But I did not really feel they did that. But it could be something they [nurse and doctor] did before I came in, I do not know. Female, 71 y.

### Self-efficacy

59% of PRO-EVAL-P respondents agreed that the questions were easy to understand while 36% noted that one or a few questions were challenging to understand. A group of 5% found several questions challenging (Fig. 1c).

Most interviewed patients described having access to electronic devices and the ability to respond. A few found the questions challenging, describing them as overly academic and complicated to comprehend. One female respondent with a long-term cardiac disease found out she had misinterpreted questions, which affected her confidence in accurately responding in the future.

Yeah, yeah, it was fine if you understand how to answer them correctly. I mean, I think it’s written by someone who knows about food, diet, and heart disease, and all that. It’s just difficult to tick a right box [put a mark in the right place]. Female, 62 y.

Some interviewees were cognitively challenged, as their memory had been affected by their disease and treatment.

They [the questionnaires] come too quickly because when you’ve just been through this, you can’t remember, and I’ve filled it out to the best of my ability, but I simply can’t recall. I can’t remember anything at all. I mean, I was at the doctor’s the other day and I couldn’t remember at all that I had such a questionnaire. Female, 56 y.

In addition, long recall periods in the questionnaire caused confusion among some patients, who were unsure which symptoms to report—those experienced before or after the treatment.

## Discussion

This study explored patients’ acceptability of the PRO-Heart-DK questionnaire using TFA, aiming to enhance the implementation of PROM in cardiac rehabilitation. The findings revealed an overall high acceptability of the PROM, but also highlighting patients’ varied emotional responses and differing perceptions regarding the questionnaire relevance and utility. These insights should be considered when revising the PRO-Heart-DK or when adapting it to other settings.

A key finding of the study is that many patients perceived the questionnaire as a valuable preparation tool that supported communication with HCPs, as long as the PROM responses were properly addressed by the HCP in the consultation. Our findings are in line with previous studies of patient experiences with PROM conducted in other clinical areas e.g [34–37]. indicating that IHD patients do not differentiate substantially when it comes to their perception of PROM. Furthermore, the use of PROM also provided a sense of safety and made individuals feel at the center of their care, which is promising as it is in accordance with international recommendations [4].

A subset of patients found it emotionally distressing to be confronted with certain topics of the PROM. Especially, the questions regarding sexual/intimacy problems caused reactions. This is interesting, as sexual dysfunction is a common problem among cardiovascular patients [38] and the topic is regarded as important in a cardiac rehabilitation setting. The presence of a potentially sensitive topic like this in a PROM legitimizes both patients and HCPs to bring it up in the conversation, as shown in studies in other clinical areas [39, 40]. Nonetheless, our study suggests that asking about it can be a significant issue, with some patients perceiving it inappropriate and/or struggling to see its relevance. Modifications were made in the PRO-Heart-DK after this feasibility study, providing context for the sexuality/intimacy questions and allowing patients to skip the topic if they perceived it as irrelevant. However, the impact of these changes on patient acceptability is yet to be assessed.

While the PRO-Heart-DK can be considered relatively lengthy with its 76 items, most patients nonetheless found the time to complete it acceptable which is likely related to their perception of relevance of the questions [41] and/or their motivation to contribute. Although readability-scores of most of the items corresponded to ‘easy’ or ‘middle’ readability, some items were more complex [42] which may contribute to the explanation of why some of the participants in our study found it difficult to comprehend all questions, indicating a need to use more plain language. Altering the readability can, however, be a problem as the PRO-Heart-DK developers aimed to include already developed and validated items/scales when compiling the questionnaire with the aim of securing item validity and to use the data also for quality improvement and research purposes [19]. The wording of these items/scales cannot be changed, at least not without renewed, resource-demanding validation processes. This issue is likely common in the selection of PROMs intended for multi-purpose application (clinical practice *and* research) also in an international perspective and raises the issue of which needs and purposes that should be prioritized—usability in clinical practice with a broad variety of patients, or fulfilling research demands such as validation and standardization—and to this, there is no standard solution.

Our data also suggested other issues with comprehensiveness and perceived relevance. First, several of the interviewees had difficulties recalling information about the purpose of PROM. Although all patients received written information about PROM, verbal communication seemed more effective. The understanding of the purpose and mechanisms of PROMs holds significance as it essentially represents the face validity of the intervention [24]. The findings indicate a need for improved communication to secure that patients understand why they are invited to use PROM, and is a potential topic for future research. Second, data suggested issues related to recall periods that stretched to before the patient’s onset of disease, which for some patients made it difficult to choose a ‘true’ response. Further, our study suggested recall issues related to cognitive impairment due to the IHD, especially memory loss post-incident. Both issues presents a significant challenge [43] as it can impact both the acceptability and the response quality. Exploring the recall period’s influence on ischemic patients’ responses in cardiac rehabilitation appears relevant for future enhancement of PROM utility in this patient group.

### Strengths and limitations

The data in this study stems from a feasibility study embedded in real-life practice. Further, it combines the strengths of both qualitative and quantitative methodologies [44, 45] and uses a theoretical framework to structure and organize analyses [46] which enhances generalizability across settings. However, we did not use the TFA prospectively, e.g. in development of the surveys and interview-guide [24], thus we did not have both types of data for all seven constructs. Further, there are no defined thresholds for acceptability and as a consequence it will be up to the individual researchers to make a judgement about the ‘level’ of acceptability. A potential bias is that our data may not reflect the experiences of the whole spectra of patients with a potential need for cardiac rehabilitation as research shows that patients that are more resourceful are selected and willing to participate in cardiac rehabilitation [47, 48].

It is a limitation that we did not have access to data describing more characteristics of the participants compared to a background population, with the exception of gender, where there were slightly more women participating in our study compared to the 25% women who participate in cardiac rehabilitation overall [49]. Furthermore, the PFF seems best suited as an evaluation survey for patients who have had regular consultations before PROM was introduced, which enables patients to compare and evaluate usefulness of PROM vs. no PROM. Finally, the context is important in deciding whether or not findings may have meaning in other settings [50]. This study was situated in cardiac rehabilitation settings in Denmark, and it should be evaluated by the reader whether findings are transferable to other contexts where PROMs are implemented as part of clinical practice.

## Conclusion

This mixed-method study conducted in hospital and municipality settings in Denmark indicated an overall high patient acceptability of a national PROM within cardiac rehabilitation as a majority of the patients found the PROM to be effective in terms of improving the consultation at the same time as the perceived effort to complete it was acceptable to most. The patients’ varied experiences highlighted factors that both contribute to and may impede the acceptability of the PRO-Heart-DK. The study emphasizes the significance of securing questionnaire relevance, comprehensiveness, and structure in the development process. In the use of PROMs in clinical practice, attention should be paid to giving adequate information about the purpose of PROM and using the answers in the consultation, as well as being attentive to possible emotional reactions. These elements play an important role in shaping patients’ perceptions of the PROM’s value and may influence their motivation for future engagement.

## Electronic supplementary material

Below is the link to the electronic supplementary material.

Supplementary Material 1

Supplementary Material 2

Supplementary Material 3

Supplementary Material 4

## Data Availability

The quantitative (survey) datasets used during the current study are available from the corresponding author on reasonable request. The qualitative (interview) data are not available, as they are regarded as sensitive personal data and not anonymized.

## Abbreviations

HCP: Healthcare Professional
IHD: Ischemic heart disease
PFF: Patient Feedback Form
PRO-EVAL-P: The PRO Evaluation Questionnaire
PROM: Patient-reported outcome measure
PRO-Heart-DK: A new, nationally developed PROM for cardiac rehabilitation in Denmark
