# Supplementary material for: Patients’ acceptability of a patient-reported outcome measure in cardiac rehabilitation (the PRO-Heart-DK)—a mixed methods study using the Theoretical Framework of Acceptability
Source: J Patient Rep Outcomes. 2025 Mar 25;9:35. doi: 10.1186/s41687-024-00831-8 (PMC11937467; doi:10.1186/s41687-024-00831-8)
Supplement: Supplementary file 1 — Supplementary Material 1 [file 41687_2024_831_MOESM1_ESM.docx]

**Appendix 1:** Domaines, scales and items in the PRO-Heart-DK, full questionnaire, tested in the period December 2019-October 2020.

| **Domaine** | **Topic** | **Scale/item** |
| --- | --- | --- |
| Angina + dyspnea | Angina | In-house made item “Have you ever had chest pain or felt pressure over your chest?” and if ‘yes’, followed by SAQ-7 |
|  | Dyspnea/breathing difficulties/breathlessness | HeartQoL |
| Mental wellbeing | Depression | HeartQoL, WHO-5, MDI-2 |
|  | Anxiety | ASS-2 |
|  | Stress | WHO-5 |
| Health related quality of life | Health related quality of life | HeartQoL, SAQ-7, SF-1 |
| Functioning | Physical functioning | HeartQoL, SAQ |
|  | Cognitive functioning | Items from SCL-92 |
|  | Intimacy/sexuality | Life with a heart disease (Danish questionnaire) |
|  | Sleep/fatigue | HeartQoL, Items from SCL-92 |
| Social wellbeing | Support from network | Item from the DenHeart study questionnaire / The Danish National Health Survey |
|  | Loneliness | Item from the DenHeart study questionnaire / The Danish National Health Survey |
| Coping with illness | Coping with illness | PAM |
|  | Medication / adjustment of medication | SAQ-7 |
| Risk factors | Smoking | In-house made items based on variables in the Danish Cardiac Rehabilitation Database |
|  | Alcohol | In-house made items based on variables in the Danish Cardiac Rehabilitation Database |
|  | Physical exercise | In-house made items inspired by The Danish National Health Survey |
|  | Diet | HeartDiet (Danish food-frequency PROM) |
| **Abbreviations:**  SAQ: Seattle Angina Questionnaire  HeartQoL: Heart Quality of Life  WHO: World Health Organization  MDI: Major Depression Inventory  ASS: Anxiety Symptom Scale  SF: Short Form  SCL: Symptom CheckList  PAM: Patient Activation Measure | | |

Please note: Freely translated for the purpose of this Appendix. Source: The Danish Health Data Authority (2019). Report: PRO for cardiac rehabilitation. Report from the workshops for development of PRO-tools in cardiac rehabilitation (available (in Danish only) at <https://pro-danmark.dk/da/pro-emner/hjerterehabilitering>).
