# Supplementary material for: Patients’ acceptability of a patient-reported outcome measure in cardiac rehabilitation (the PRO-Heart-DK)—a mixed methods study using the Theoretical Framework of Acceptability
Source: J Patient Rep Outcomes. 2025 Mar 25;9:35. doi: 10.1186/s41687-024-00831-8 (PMC11937467; doi:10.1186/s41687-024-00831-8)
Supplement: Supplementary file 2 — Supplementary Material 2 [file 41687_2024_831_MOESM2_ESM.docx]

# Appendix 2

| Table 1. Responses to the PRO Evaluation Questionnaire (PRO-EVAL-P) patient survey (n=105) | | | | | | | |
| --- | --- | --- | --- | --- | --- | --- | --- |
| Question* | **Response option (n / %**)** | | | | | | |
| How relevant were the questions for your cardiac rehabilitation process? | 1  Not relevant at all | | 2 | 3 | 4 | 5  Very relevant | Missing |
|  | 1 (1.0%) | | 8 (7.6%) | 32 (30.5%) | 33 (31.4%) | 31 (29.5%) | 0 (0%) |
| How easy/difficult was it for you to respond to the questionnaire? | 1  Very  difficult | | 2 | 3 | 4 | 5  Very easy | Missing |
|  | 0 (0%) | | 10 (9.5%) | 31 (29.5%) | 35 (33.3%) | 29 (27.6%) | 0 (0%) |
| Question* |  | | | **Response option (n / %**)** | | | |
| Were there any questions that were difficult to understand? |  |  | | No | Yes, but only one or a few | Yes, several | Missing |
|  |  |  |  | 62 (59.0%) | 38 (36.2%) | 5 (4.8%) | 0 (0%) |
| Were there lacking any questions about important subjects related to your life with a cardiac disease or the rehabilitation? |  |  | | No | Yes, to some degree | Yes, to a high degree | Missing |
|  |  |  |  | 90 (85.7%) | 11 (10.5%) | 1 (1.0%) | 3 (2.9%) |
| Were there any questions in the questionnaire that you did not like to respond to? |  |  | | No | Yes, but only one or a few | Yes, several | Missing |
|  |  |  |  | 83 (79.1%) | 21 (20.0%) | 0 (0%) | 1 (1.0%) |
| *Questions were translated (not validated translation) from Danish for the purpose of presenting data in this article.  **Percentages are rounded to one decimal and might therefore not add up to 100%. | | | | | | | |

| Table 2. Responses to the Patient Feedback Form (PFF) patient survey (n=119) | | | | | |
| --- | --- | --- | --- | --- | --- |
| Question | **Response option (n / %*)** | | | | |
|  | Strongly Agree | Agree | Disagree | Strongly Disagree | Missing |
| Completing the questionnaire made it easier for me to remember my symptoms and side effects when I met with my healthcare professional | 30 (25.2%) | 68 (57.1%) | 15 (12.6%) | 3 (2.5%) | 3 (2.5%) |
| Completing the questionnaire improved discussions with the healthcare professional | 43 (36.1%) | 64 (53.8%) | 8 (6.7%) | 0 (0%) | 4 (3.4%) |
| The healthcare professional used information from the questionnaire for my care | 56 (47.0%) | 56 (47.0%) | 4 (3.4%) | 0 (0%) | 3 (2.5%) |
| The quality of my care was improved because of the questionnaire | 30 (25.2%) | 72 (60.5%) | 9 (7.6%) | 1 (0.8%) | 7 (5.9%) |
| Communication with the healthcare professional was improved because of the questionnaire | 44 (37.0%) | 60 (50.4%) | 8 (6.7%) | 0 (0%) | 7 (5.9%) |
| Completing the questionnaire made me feel more in control of my own care | 50 (42.0%) | 60 (50.4%) | 8 (6.7%) | 0 (0%) | 1 (0.8%) |
| I would recommend completing the questionnaire to other patients | 69 (58.0%) | 42 (35.3%) | 3 (2.5%) | 1 (0.8%) | 4 (3.4%) |
| I would like to continue responding to the questionnaire in the future | 53 (44.5%) | 56 (47.0%) | 4 (3.4%) | 4 (3.4%) | 2 (1.7%) |
| *Percentages are rounded to one decimal and might therefore not add up to 100%. | | | | | |
