# Supplementary material for: Patients’ acceptability of a patient-reported outcome measure in cardiac rehabilitation (the PRO-Heart-DK)—a mixed methods study using the Theoretical Framework of Acceptability
Source: J Patient Rep Outcomes. 2025 Mar 25;9:35. doi: 10.1186/s41687-024-00831-8 (PMC11937467; doi:10.1186/s41687-024-00831-8)
Supplement: Supplementary file 3 — Supplementary Material 3 [file 41687_2024_831_MOESM3_ESM.docx]

# Appendix 3: Thematic Analyses

All recorded interviews were transcribed into written form as initial familiarizing with the data [1]. The transcripts were then imported into NVivo qualitative analysis program, where data were stored and organized.

**Phase 1:** familiarizing with the data

To begin with, all interview transcripts were read and re-read by both analysts (the first and last authors). The purpose was to become familiar with all aspects of the collected empirical data. At the same time first formed impressions of the data and immediate ideas for coding were noted [1].

**Phase 2:** generating initial codes

After familiarizing, each interview was coded separately in NVivo to make sure no data was overlooked [1] the sections of text with interesting information were highlighted. Codes were ascribed to these text sections (example provided in Table 1, below) describing basic characteristic of text segments. Analytic reflections, ideas and questions related to text segments were also captured during this process by using NVivo annotations. There were 180 codes. Twenty percent of interview data (five interviews) were double coded by the first and last author, and in case of disagreements these were discussed and agreed upon. The remaining authors acted as critical peers.

**Phase 3**: searching for themes

After all data were coded, the codes with associated text extracts were transferred to a word document. Then this was printed out and papercut. In this phase the codes with text extracts that represented the same characteristics and aspects were sorted and piled into potential subthemes (Appendix 3, Figure 1)[1]. This process included ongoing discussions between authors.

**Phase 4:** Reviewing themes

In this phase, the developed subthemes were reviewed and assessed in relation to the codes and overall transcribed data [1].

**Phase 5:** Application of TFA

In this phase, the subthemes were organized and interpreted in accordance to the according to The Theoretical Framework of Acceptability (TFA) constructs. This required an iterative collaborative process between the authors, until the final interpretation was reached.

**Appendix 3,** **Table 1.** Generating codes. Data extracts with codes applied

| **Data extract** | **Coded for** |
| --- | --- |
| P 7: I do not know it 100%, but I could imagine that it is to continuously develop treatment of cardiac patients who have been exposed to something like me. That one can get wiser about different things and what might be the reason why it was as it was, or has become as it was. That’s it, I cannot 100% say what the purpose is.  P 17: But as I said, I have an opinion which is, if such questionnaires and the answers that I can give now, can help in the future towards future patients, if I can give something back in that way, that it can help that there continues to be a development also for the treatment of such patients as me. If it can help to give some feedback so you can get wiser on some points, than I also think it’s worth it right? Because I also think that it is fantastic all the treatment I have been through, that is, both since I started at the hospital, I have only praised them and I think it is absolutely fantastic what they can do today. I have always had the attitude that, of course, it can help to make it better in the future, because there is constant research into what we can do better right? | Do not know what the purpose of PROM is  Probably used for development of treatment for cardiac patients  It is worth it if questionnaires can help other patients, and healthcare professionals with continuous development of treatment.  A way to give back for fantastic treatment that was received  Motivated to engage because it can help with research and advancements |

**Appendix 3, Figure 1.** Searching for themes, initial thematic map showing initial subthemes and how these connect


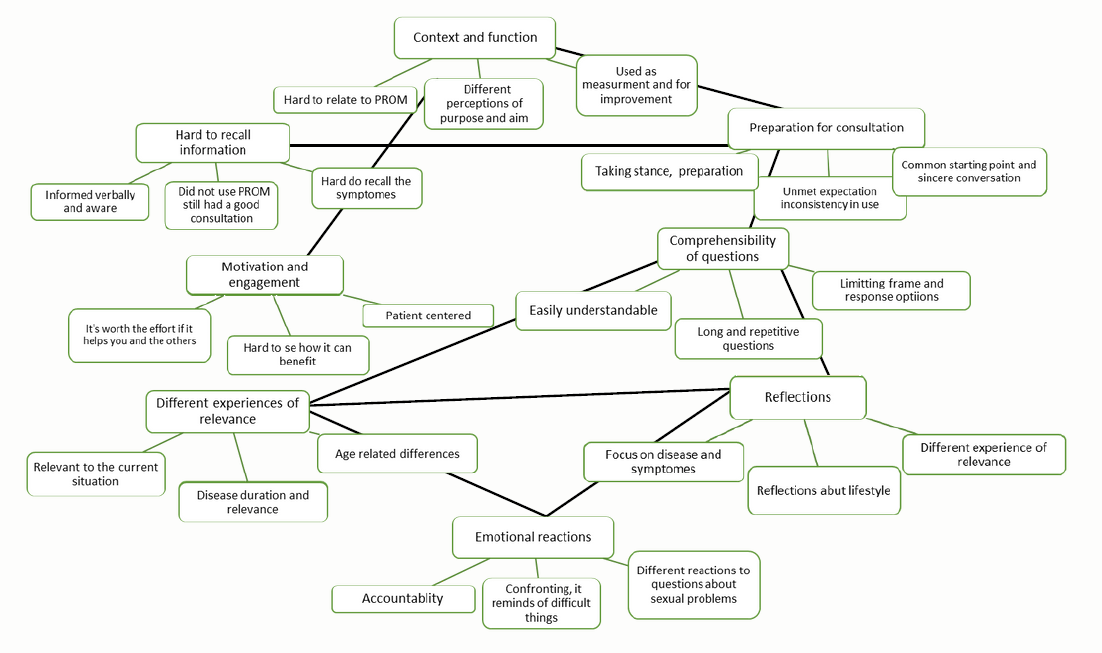


1. Braun, V., & Clarke, V. (2006). Using thematic analysis in psychology. *Qualitative Research in Psychology, 3*(2), 77-101, doi:10.1191/1478088706qp063oa.
