# Supplementary material for: Patients’ acceptability of a patient-reported outcome measure in cardiac rehabilitation (the PRO-Heart-DK)—a mixed methods study using the Theoretical Framework of Acceptability
Source: J Patient Rep Outcomes. 2025 Mar 25;9:35. doi: 10.1186/s41687-024-00831-8 (PMC11937467; doi:10.1186/s41687-024-00831-8)
Supplement: Supplementary file 4 — Supplementary Material 4 [file 41687_2024_831_MOESM4_ESM.docx]

**Appendix 4:** Application of the Theoretical framework of acceptability (Sekhon et al. 2017)

| Theoretical Frame-work of Acceptability constructs | Definition | Interpretation of the concepts | Patient Feedback Form (PFF) | PRO Evaluation Questionnaire (PRO-EVAL-P) | Subthemes identified in inductive analysis – deductively applied to the TFA |
| --- | --- | --- | --- | --- | --- |
| Overall acceptability |  | Patients’ general comments/ responses regarding their willingness to engage in and recommend the use of PROM | I would recommend completing the questionnaire to other patients  I would like to continue responding to the questionnaire in the future |  | Altruistic motivation |
| Affective attitude | How an individual feels about the intervention | Patients’ reactions to the questionnaire structure, content and use of data, e.g. what they experienced as disturbing |  | Were there any questions in the questionnaire that you did not like to respond to? | Confronting to respond and discouraging results  Distress, reluctance to respond, sadness loss of function  Need to defend one self and accountability |
| Burden | The perceived amount of effort that is required to participate in the intervention | Patients’ perceived and encountered challenges when responding to the questionnaire |  | How easy/difficult was it for you to respond to the questionnaire? | Easy to reply  Limited, too long, |
| Ethicality | The extent to which the intervention has good fit with an individual’s value system | Features of the questionnaire in regards to patients’ needs related to their disease |  | How relevant were the questions for your cardiac rehabilitation process?  Were there lacking any questions about important subjects related to your life with a cardiac disease or the rehabilitation? | Different reactions to intimacy questions  Rigid structure |
| Intervention Coherence | The extent to which the participant understands the intervention and how it works | Patients’ insights, assumptions and knowledge about the PROM |  |  | Different assumptions about its use  Lack of awareness hard to relate to PROM |
| Opportunity Cost | The extent to which benefits, profits or values must be given up, by engaging in the intervention | Patients’ experiences in regards to equipment, time, and the general requirements to respond |  |  | Time consuming  Help from spouse, neighbor and/or children |
| Perceived Effectiveness | The extent to which the intervention is perceived as likely to achieve its purpose | Patients’ perceptions of the relevance, usefulness and value of the PROM in relation to their disease | The quality of my care was improved because of the questionnaire  Completing the questionnaire made it easier for me to remember my symptoms and side effects when I met with my healthcare professional  Completing the questionnaire improved discussions with the healthcare professional  Communication with the healthcare professional was improved because of the questionnaire  The healthcare professional used information from the questionnaire for my care  Completing the questionnaire made me feel more in control of my own care |  | Useful tool, it forges reflections and change  Help to take a stand on topics but not all patients need it  Common starting point and sincere communication  Variation in how data was used by HCPs  Content not relevant for all patients |
| Self-efficacy | The participants’ confidence that they can perform the behaviour(s) required to participate in the intervention | Patients’ experiences with completing the PROM, e.g. sense of having the ability, understanding the questions |  | Were there any questions that were difficult to understand? | Difficult to understand for some  Cognitive challenges |
